# Acupuncture for swallowing disorder after recovery from COVID-19: A protocol for systematic review and meta analysis

**DOI:** 10.1097/MD.0000000000032491

**Published:** 2023-03-31

**Authors:** Yao Xiao, Yueqi Lin, Qiqi Chen, Runyi Wang, Zuming Li, Daman Chen, Yuxin Huang, Guiyuan Peng

**Affiliations:** a Guangzhou University of Chinese Medicine, Guangzhou, China; b Department of Otolaryngology-Head and Neck, Guangdong Province Traditional Chinese Medical Hospital, Guangzhou, China.

**Keywords:** acupuncture, coronavirus disease 2019, protocol, swallowing disorder

## Abstract

Swallowing disorder is a common sequela after recovery from COVID-19. Acupuncture is an important traditional therapy for treating swallowing disorder. However, the efficacy of acupuncture for swallowing disorder after recovery from COVID-19 lacks evidence-based medicine.

**Methods:**

All randomized controlled trials of acupuncture for swallowing disorder after recovery from COVID-19 will be retrieved and collected from December 2019 to November 2022 with no language restrictions. PubMed, EMBASE, Cochrane Library, Web of Science, China National Knowledge Infrastructure Database, Chinese Biomedical Database, Chinese Science and Technology Journal Database (VIP), and the Wanfang Database will be searched. Two researchers will independently select studies, extract data, and evaluate study quality. The Cochrane risk of bias tool for randomized trials will be used to assess the risk of bias in the included studies. Statistical analyses will be performed using Review Manager version 5.3.

**Results:**

This study will provide a high-quality and convincing assessment of the efficacy and safety of acupuncture for swallowing disorder after recovery from COVID-19 and will be published in peer-reviewed journals.

**Conclusion:**

Our findings will provide a reference for future clinical decisions and guidance development.

## 1. Introduction

Coronavirus disease 2019 (COVID-19) is a new acute respiratory infectious disease, which has been spreading worldwide since it was declared an international public health emergency by the World Health Organization on January 30, 2020, and is a serious threat to human health. It has overburdened the health systems of most countries and caused major deaths and huge economic losses.^[1]^ Because of the novel coronavirus’s damage to multiple organs of the human body, a considerable proportion of COVID-19 patients will develop various sequelae symptoms, including swallowing disorder, for a long time after recovery.^[2]^ As more patients began to recover in the ICU, swallowing disorder became more apparent as a key focus of rehabilitation. Studies have shown that many COVID-19 patients still have persistent swallowing disorder after discharge, and the symptoms are obvious, but they can be easily ignored.^[3,4]^ In Italy, the prevalence of swallowing disorder in those requiring inpatient rehabilitation was high, with 90% of 50 patients admitted to their COVID-19 rehabilitation center requiring a modified diet or tube feeding on admission.^[5]^ One published European paper reported 41 COVID-19 non-intubated hospitalized patients. Eight patients (20%) presented with swallowing disorder at the 6-month follow-up, and only 2 still self-reported swallowing difficulties.^[6]^ Swallowing disorder refers to the process by which food cannot be safely and effectively transported to the stomach due to damage to the structure and/or function of organs, such as the mandible, lips, tongue, soft palate, throat, and esophagus, etc. It may cause a series of serious consequences such as aspiration pneumonia, dehydration, malnutrition, disability, and increased mortality. Therefore, relieving swallowing disorder in COVID-19 patients is helpful in protecting their lives and improving their quality of life. Unfortunately, the current modern medicine for the treatment of swallowing disorder, mainly to reduce aspiration, improve swallowing function and improve the nutritional status of patients, there is no targeted and exact treatment.

Acupuncture is a well-known external treatment used in traditional Chinese medicine. It has a history of more than 2000 years in China and has been widely used in 183 countries worldwide. This is an important traditional therapy for swallowing disorder. It has been confirmed that acupuncture has unique advantages in the treatment of swallowing disorder, and its curative effect has been put into practice in preclinical and clinical trials.^[7–9]^ Studies have shown that the mechanism by which acupuncture improves swallowing disorder may be related to increased hyoid movement displacement and shortened pharynx administration time.^[10]^ One study suggested that electroacupuncture at acupoints Fengfu and Lianquan promotes swallowing activity.^[11]^ Combined with chemical genetics, electromyography recording, and immunofluorescence staining, it has been shown that electroacupuncture stimulation of acupoint lianquan can activate paraventricular hypothalamus neurons and regulate human swallowing function.^[12]^ Thus, acupuncture can significantly improve the swallowing function of patients with swallowing disorder, with quick effects, few adverse reactions, convenient operation, low cost, and diversity of therapy.^[13–16]^ During the epidemic of COVID-19, acupuncture has been used as a supplementary treatment of COVID-19 in China, and its curative effect has been proved by research.^[17–21]^ Therefore, acupuncture may be an effective and safe method for the treatment of swallowing disorder in COVID-19 patients.^[22]^

To date, there is no high-quality evidence on acupuncture in the treatment of swallowing disorder after recovery from COVID-19. Therefore, we designed this study to better understand the effectiveness and safety of acupuncture therapy for swallowing disorder following recovery from COVID-19.

## 2. Method and analysis

### 2.1. Objectives and registration

This systematic review aimed to evaluate the efficacy of acupuncture in the treatment of swallowing disorder after recovery from COVID-19. Our proposal was registered with PROSPERO (registration number: CRD42022371868). We strictly followed the recommendations outlined in the Cochrane Handbook of Systematic Reviews of Interventions, as well as the preferred reporting items in the Guidelines for Preferred Reporting Items for Systematic Reviews and Meta-Analysis Protocols Statements. All steps in this systematic review will be performed in accordance with the Cochrane Handbook. If modifications are required, the study protocol will be updated to include changes throughout the study process.

### 2.2. Inclusion and exclusion criteria

#### 1.2.2. Types of studies.

This study will include randomized controlled trials of acupuncture alone or in combination with other interventions for the treatment of swallowing disorder after recovery from COVID-19. We will focus on the Chinese and English literature related to clinical studies, excluding non-randomized controlled trials, retrospective studies, reviews, conference abstracts, case reports, animal studies, and literature without data.

#### 2.2.2. Participants.

There are no restrictions on sex, age, occupation, race, or country for patients who have been diagnosed with COVID-19 and are now working with post-recovery patients with swallowing disorder sequela. Patients with a history of swallowing disorder prior to COVID-19 infection and other severe mental, cardiovascular, and cerebrovascular diseases will be excluded, and the diagnosis of swallowing disorder included Chinese or international diagnostic criteria.

#### 2.2.3. Types of interventions.

The intervention measures of the treatment group were acupuncture, while those of the control group were fake acupuncture, acupoint application, moxibustion, routine rehabilitation training, and conventional Western medicine treatment.

#### 2.2.4. Tape of outcomes.

The main outcomes of this meta-analysis are as follows: water swallow test, standardized swallowing assessment, video fluoroscopic swallowing study, and fiberoptic endoscopic examination of swallowing. The secondary results were standard of life, symptoms, and adverse outcomes.

### 2.3. Research the retrieval method of identification

Randomized controlled trials were extracted from PubMed, EMBASE, Cochrane Library, Web of Science, China National Knowledge Infrastructure Database, Chinese Biomedical Database, Chinese Science and Technology Journal Database, and the Wanfang Database from December 2019 to November 2022. All randomized controlled trials of acupuncture for swallowing disorder after recovery from COVID-19 were searched for and collected. Table 1 presents the retrieval strategy using PubMed as an example. Similar strategies will be applied to other databases after adjustment. No language restrictions were imposed.

**Table 1:** Search strategy used in PubMed database.

#1 Swallowing Disorder
OR Swallowing Disorders OR Dysphagia
OR Deglutition Disorder OR Deglutition Disorders
OR Swallowing Dysfunction
#2 COVID-19 OR COVID 19
OR SARS-CoV-2 Infection OR Infection, SARS-CoV-2
OR SARS CoV 2 Infection OR SARS-CoV-2 Infections
OR 2019 Novel Coronavirus Disease OR 2019 Novel Coronavirus Infection
OR 2019-nCoV Disease OR 2019 nCoV Disease
OR 2019-nCoV Diseases OR Disease, 2019-nCoV
OR COVID-19 Virus Infection OR COVID-19 Pandemics
OR COVID19 Pandemic OR 2019-nCoV Infections
OR COVID-19 Virus Diseases OR SARS Coronavirus 2 Infection
OR Disease2019, Coronavirus
#3 Acupuncture OR Needling OR Electroacupuncture OR Warm acupuncture
#4 Randomized controlled trial OR clinical study OR Clinical Trial OR Controlled study OR Controlled Trial OR Random*Control* study OR random* Control* Trial
#5 #1 AND #2 AND #3 AND #4

### 2.4. Data collection

#### 2.4.1. Data filtering.

**Figure 1.**
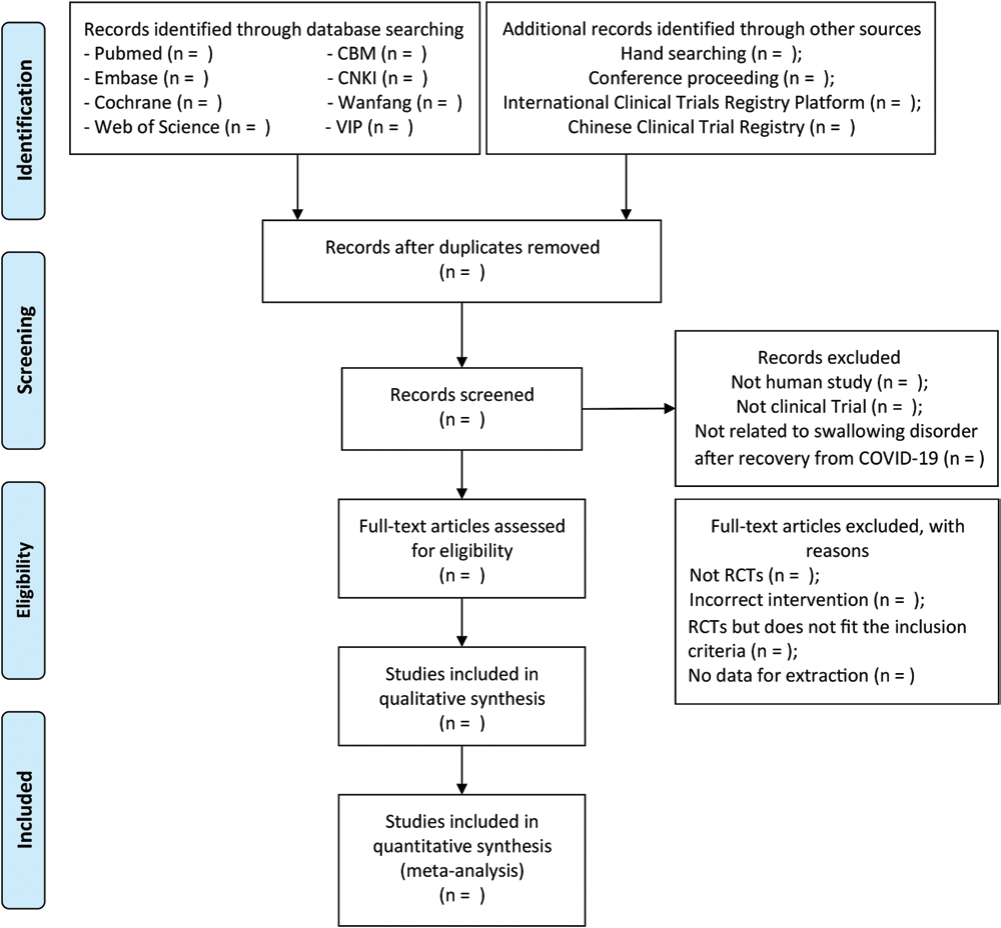
PRISMA flow chart of study selection process. PRISMA = Preferred Reporting Items for Systematic Reviews and Meta-Analysis Protocols.

All retrieved documents will be imported into NoteExpress software (version 3.0), and duplicate documents will be excluded. First, 2 researchers (X.Y. and L.Y.Q.) will conduct an initial screening by reading the titles and abstracts based on predetermined inclusion and exclusion criteria. The remaining articles will be read in full after downloading for further screening. All exclusions will be provided for the appropriate reasons. Any disagreements that arose during this process will be resolved by a third researcher (C.Q.Q). The process of filtering the selection is shown in the Preferred Reporting Items for Systematic Reviews and Meta-Analysis Protocols flowchart in Figure 1.

#### 2.4.2. Data extraction and management.

The methodological quality of the RCTS and risk of bias will be independently assessed by 2 investigators (X.Y. and L.Y.Q.) using the Cochrane bias risk assessment tool. The researchers will evaluate 7 areas: random sequence generation, assignment hiding, participant, and person blindness, outcome evaluation blindness, incomplete outcome data, selective reporting, and other bias. Based on the research evaluation of these 7 areas, they are classified into “low risk,” “high risk,” and “undefined risk” bias. If there is disagreement between the evaluation results of the 2 research members, the third researcher (C.Q.Q.) will decide the final evaluation result and display it in the form of a chart.

#### 2.4.3. Processing lost data.

If there is any missing or unclear information in the RCT literature, we will attempt to contact the corresponding author to obtain the corresponding information. If the information is incomplete, then the literature will be deleted.

### 2.5. Statistical analysis

Meta-analysis of the literature was performed using the RevMan5.3 statistical software (*P* < .5 was statistically significant). Two researchers (X.Y. and L.Y.Q.) are responsible for data extraction, input, and calculation and a third researcher (C.Q.Q.) is responsible for data validation. We will use the mean deviation or standard mean deviation of the 95% confidence interval (CI) as an effect measure for continuous data. Risk ratios and 95% CI were used to analyze dichotomous results. *I*^2^ statistics were used to detect the clinical heterogeneity. Heterogeneity will be assessed using the chi-square test and Higgins *I*^2^ test. If there was no significant heterogeneity (*I*^2^ < 50%, *P* > .1), a fixed-effects model was used. The random effects model is used for meta-analysis if *P* < .1 and *I*^2^ > 50%. Significant clinical heterogeneity will be addressed by subgroup analysis, sensitivity analysis, or descriptive analysis.

### 2.6. Evaluation of heterogeneity

Heterogeneity was assessed using Cochrane and *I*^2^ tests. If each study in the subgroup was statistically homogeneous (*P* ≥ .5, *I*^2^ ≤ 50%), the heterogeneity evaluation could be ignored, and the fixed-effect model was used for meta-analysis. If *P* < .5 and *I*^2^ > 50%, there was a large heterogeneity among the included studies.

### 2.7. Assessment of reporting deviations

When more than 10 studies were included in the meta-analysis, we used RevMan5.4.1 software to draw funnel plots and Egger checks to assess report bias.

### 2.8. Subgroup analysis and sensitivity analysis

The clinical or methodological heterogeneity of included RCTs was the main reason for the statistically significant heterogeneity. If heterogeneity between studies is high, a subgroup analysis will be performed. Possible sources of heterogeneity will be investigated based on duration of treatment, sex, age, ethnicity, study quality, and bias risk analysis. If the heterogeneity of the results was high (*I*^2^ > 50%), sensitivity analysis was performed to test the robustness of the study conclusions. According to the assessment results of bias risk and methodological quality, we will exclude low-quality RCTs to conduct sensitivity analysis to ensure the stability of the conclusions.

### 2.9. Grade the quality of evidence

We will use the Recommended Grading Assessment, Development and Evaluation Grading (GRADE) system to assess the overall quality of the included research evidence, and classify the results according to the rating criteria as “high,” “medium,” “low,” and “very low.”

### 2.10. Ethics and communication

This study did not involve personal data related to the participants and therefore did not require ethical approval; our research results will be shared and disseminated through conference reports and peer-reviewed publications.

## 3. Discussion

Acupuncture can effectively relieve dysphagia^[23]^ and promote post-operative recovery. Simultaneously, acupuncture as a long history traditional Chinese medicine treatment has the advantages of few adverse events and no drug dependence, and combining modern medical technology and theory has achieved good curative effects.^[24]^ At present, there are few systematic studies on acupuncture for the treatment of swallowing disorder after recovery from COVID-19. It is necessary to conduct a systematic review to establish convincing evidence to evaluate the effectiveness and safety of acupuncture for swallowing disorder after recovery from COVID-19. Therefore, we will adopt a more rigorous systematic evaluation method to provide evidence-based data for the treatment of swallowing disorder after recovery from COVID-19 using acupuncture and provide new ideas and methods for the treatment and research of swallowing disorder after recovery from COVID-19.

## Author contributions

**Conceptualization:** Qiqi Chen.

**Data curation:** Yao Xiao, Qiqi Chen.

**Funding acquisition:** Guiyuan Peng.

**Investigation:** Yao Xiao, Yueqi Lin, Runyi Wang.

**Methodology:** Yueqi Lin.

**Resources:** Runyi Wang, Daman Chen, Yuxin Huang.

**Software:** Runyi Wang, Zuming Li, Daman Chen, Yuxin Huang.

**Supervision:** Qiqi Chen.

**Validation:** Zuming Li, Daman Chen.

**Writing – original draft:** Yao Xiao, Yueqi Lin, Qiqi Chen.

**Writing – review & editing:** Yao Xiao, Yueqi Lin, Qiqi Chen.
